# Comparison of end-tidal carbon dioxide levels with cardiopulmonary resuscitation success presented to emergency department with cardiopulmonary arrest.

**DOI:** 10.12669/pjms.301.4024

**Published:** 2014

**Authors:** Emine Akinci, Hayri Ramadan, Yucel Yuzbasioglu, Figen Coskun

**Affiliations:** 1 Emine Akinci, Emergency Medicine Department, Kecioren Training and Research Hospital, Ankara, Turkey.; 2 Hayri Ramadan, Emergency Medicine Department, Ankara Training and Research Hospital, Ankara, Turkey.; 3 Yucel Yuzbasioglu, Emergency Medicine Department, Ankara Training and Research Hospital, Ankara, Turkey.; 4 Figen Coskun, Emergency Medicine Department, Ankara Training and Research Hospital, Ankara, Turkey.

**Keywords:** PetCO2, Cardiopulmonary Resuscitation

## Abstract

***Objective:*** To measure end-tidal carbon dioxide pressure (PetCO2) in preset interval in order to evaluate the efficiency of cardiopulmonary resuscitation (CPR) performed on patients in cardiopulmonary arrest, evaluate the validity of PetCO2 in predicting the mortality and finally assess the PetCO2 levels of the patients in cardiopulmonary arrest based on the initial presenting rhythm.

***Methods: ***This prospective study was conducted at the Ankara Training and Research Hospital on patients who presented with cardiopulmonary arrest. Standard ACLS (Advanced Cardiac Life Support) protocols were performed. Patients were categorized in two groups based on their rhythms as Ventricular Fibrillation and Asystole. Patients’ PetCO2 values were recorded.

***Results: ***PetCO2 levels of the Return of Spontaneous Circulation (ROSC) group in the 5th, 10th, 15th and 20th minutes were significantly higher compared to the exitus group (p<0.001). In distinguishing ROSC and exitus, PetCO2 measurements within 5-20 minute intervals showed highest performance on the 20th and lowest on the 5th minutes.

***Conclusion: ***PetCO2 values are higher in the ROSC group. During the CPR, the most reliable time for ROSC estimation according to PetCO2 values is 20th minute. None of the patients who had PetCO2 levels less than 14 mmHg survived.

## INTRODUCTION

In order to standardize the quality of cardiopulmonary resuscitation (CPR) and simultaneous monitoring, certain mechanical or physiological parameters are considered.^1^^,^^2^ Discovery of positive relationship between the cardiac output and end-tidal carbondioxide pressure (PetCO2) has led to use of capnography during cardiopulmonary resuscitation.^3^^,^^4^ During cardiac arrest the partial pressure of end-tidal carbon dioxide falls to very low levels, reflecting the very low cardiac output achieved with cardiopulmonary resuscitation. When ventilations are provided without chest compressions during CPR, the PetCO2 levels reach down to zero after some time. Increase in pulmonary perfusion following chest compressions result in increase in PetCO2 levels, which is an indicator of effective CPR. Rapid increase in PetCO2 levels also indicates return of spontaneous circulation (ROSC).^5^^,^^6^

In 2010, AHA (American Heart Association) guidelines advised the use of quantitative waveform capnography based on PetCO2 values in adult cardiac arrests as Class-1 in determining CPR quality and ROSC.^7^ In this study, we aimed to measure end-tidal carbondioxide pressure (PetCO2) in preset interval in order to evaluate the efficiency of cardiopulmonary resuscitation (CPR) performed on patients in cardiopulmonary arrest, evaluate the validity of PetCO2 in predicting the mortality and finally assess the PetCO2 levels of the patients in cardiopulmonary arrest based on the initial presenting rhythm.

## METHODS

The study was conducted prospectively at the Ministry of Health Ankara Training and Research Hospital Emergency Department between January-August 2011 on patients presented with cardiopulmonary arrest. Ethics board approval was obtained prior to the study, and patients’ relatives were informed about the study. Advanced cardiovascular life support (ACLS) interventions included airway management, ventilatory support and treatments of bradyarrhytmias and tachyarrhythmias.

The ACLS treatment builds upon the foundation of good basic life support (BLS) which includes rapid recognition of sudden cardiac arrest, calling for help, starting immediate CPR, performing rapid defibrillation with the use of automated external defibrillators (AED), followed by more advanced procedures including advanced airway management and cardiac monitorization which are used to increase the chance for return of spontaneous circulation.^8^ We performed standard ACLS algorithms in cardiac arrest patients for whom initial ACLS treatment was given by the ambulance crews and brought into the emergency department (ED).

***Inclusion Criteria: ***Patients older than 18 years of age who suffered cardiac arrest resulting from respiratory causes (such as asthma, Chronic obstructive pulmonary disease (COPD), intoxications, foreign body aspiration, pneumonia, malignancies not in terminal stage and stroke) or cardiac (causes acute myocardial infarction, heart failure) as determined by patient history, clinical and laboratory findings were included in the study.

***Exclusion criteria:*** Trauma patients, those with severe terminal cancer patients or suffering from severe hypothermia (<30C°), those who were not brought in by ambulance and those under 18 were excluded from the study.

***Study Design: ***All ambulance crew undergo basic life support, advanced life support and trauma and resuscitation training provided by the City EMS Training Division. All ambulances carry standardized advanced life support equipment. Resuscitation team members consisted of an attending emergency physician, an emergency medicine resident and two nurses. Patients were grouped as ventricular fibrillation (VF)/ pulseless ventricular tachycardia and asystolye / pulseless electrical activity (PEA) based on their initial presenting cardiac rhythm. We measured and recorded the end-tidal CO2 levels of the patients who were intubated in the field by the ambulance crews. The measurements were performed by using EMMA (Easy Note MB85) capnometry device on arrival and every 5 minutes thereafter until either the resuscitation was ceased or the patients had return of spontaneous circulation (ROSC). 

***Study Outcome: ***Patients with palpable pulses and admitted to the intensive care unit (ICU) were considered as ROSC. Those who did not respond to Standard resuscitation protocols were pronounced dead. Concurrently, patients' demographic information such as age and gender, probable causes of arrest, arrival rhythms, whether or not Basic Life Support or ACLS was provided, and duration of CPR provided at the ED and medications were recorded on standard forms.

***Statistical Analysis: ***Data analyses were performed using SPSS 11.5 software package for Windows. Normality of the distribution of continuous variables were examined with the Shapiro Wilk Test. Defining statistics for the continuous variables were shown as mean ± standard deviation or median (lowest-highest), and categorical variables were shown as event number and percentages (%). The significance of variation in terms of averages between the groups and median values were analyzed respectively by Student T and Mann Whitney U tests. Categorical variables were evaluated either by Pearson's Chi-Square or Fisher's Exact Chi-Square tests. The presence of meaningful correlation between continuous variables was investigated with Spearman's Correlation Test. Whether PetCO2 measurements were determinant or not in distinguishing ROSC and mortality groups were analyzed by ROC analysis by calculating the area below the curve and 95% confidence intervals. “When the area under the ROC curves were statistically significant, to know which was the best cut-off points for PetCO2 to discriminate ROCS and mortality groups were calculated by Youden Index.” Additionally, in relation to this intersection point, the sensitivity, specify, positive and negative predictive values and likelihood ratios were calculated. p values less than 0.05 were considered as statistically significant. However, in order to control the possible Type-I error in multiple comparisons, Bonferroni Correction was performed. Differences between groups were analyzed by PetCO2 levels measured at 7 different time frames. The Type-I error was found as 30.2% [1-(1-0.05)^7]. In order to control the Type-I error, alpha (α) level was divided by the number of different observations and the result was 0.05/7≈0.0071. Bonferroni Correction, p<0.0071 the results were found to be statistically significant.

## RESULTS

Patients consisted of 70% males and 30% females, and the age average was 64.8. Mean duration of CPR performed in the ED was 25 minutes, and patients’ initial and final PetCO2 measurements were respectively 24.5 (3-99) and 20 (4-75) mmHg. The demographic data of the patients are given in Table-I. When arrival rhythms of the patients were considered, 77.5% had PEA or asystole, and 22.5% had VF. Between the group with VF and the group with PEA / asystole arrival rhythms there were no statistically significant differences in terms of average PetCO2 levels (p=0.519) (Table-I).

**Table-I:** Demographic and Clinical Properties of Patients

*Variables*	*n=80*		
Age (years)	64.8±12.1 (39-88)		
Gender			
Male	56 (%70.0)		
Female	24 (%30.0)		
Average transport time (min)	15 (8-22)		
Average CPR time in ambulance (min)	24.6±11.7		
Arrival Rhythm		Average PetCO2 values	p-value 0.519
VF/pulseless VT	18 (%22.5)	19.6 (13.8-39.0)*	
PEA/Asystole	62 (%77.5)	23.7 (6.8-79.4)*	
Initial PetCO_2 mm/Hg_	24.5 (3-99)*		
Final PetCO_2_	20 (4-75)*		
CPR duration (min)	25 (5-50)		
Outcome			
Exitus	56 (%70.0)		
ROSC	24 (%30.0)		

* The values in parentheses ​​are min-max values, are given as range

When the patients were evaluated in terms of their arrival rhythms, the age average of the PEA / asystole group was significantly higher than that of the VF group (p=0.043). No statistically significant difference in gender distribution, CPR duration and result was found between in the groups (p>0.05). During the patient monitoring periods, no statistically significant differences were observed in terms of PetCO2 levels between the groups (p>0.05). (Table-II)

**Table-II:** Demographic and Clinical Properties of Patients Based on Arrival Rhythm

*Variables*	*VF (n=18)*	*PEA/Asystole (n=62)*	*p-value*
Age	59.7±11.2	66.3±12.0	0.043
Gender			0.413
*Male*	14 (%77.8)	42 (%67.7)	
*Female*	4 (%22.2)	20 (%32.3)	
CPR Duration	25 (10-50)	25 (5-50)	0.650
Outcome			0.161
*Exitus*	15 (%83.3)	41 (%66.1)	
*ROSC*	3 (%16.7)	21 (%33.9)	
PetCO_2_ 0th min	22 (15-54)	25 (3-99)	0.940
PetCO2 5th min	20.5 (16-32)	22 (4-84)	0.764
PetCO2 10th min	20 (12-45)	22 (4-85)	0.586
PetCO2 15th min	18 (12-46)	21 (6-60)	0.497
PetCO2 20th min	16.5 (10-40)	20.5 (4-69)	0.526
PetCO2 25th min	16 (10-40)	20 (8-60)	0.268
PetCO2 30th min	18 (14-20)	17 (10-66)	0.717
PetCO2 35th min	14 (13-23)	18 (8-29)	1.000
PetCO2 40th min	14 (13-24)	17 (10-18)	0.905

**Fig.1:**
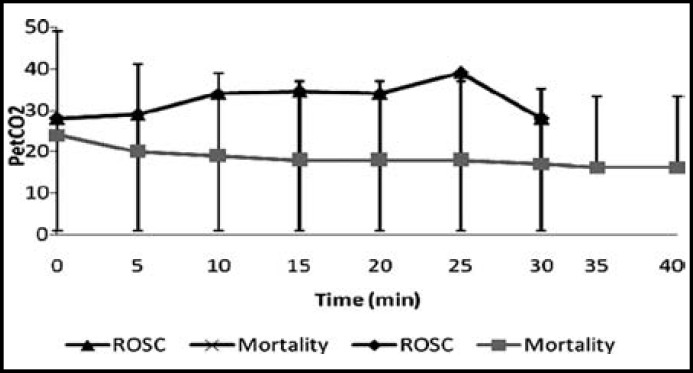
PetCO2 values in ROSC and Exitus groups

PetCO2 values in ROSC and Mortality groups are given Fig.1. Between the ROSC and mortality groups, no statistically differences existed as per Bonferroni Correction in terms of PetCO2 levels at 0th, 25th and 30th minutes (p:0.058, p:0.033, p:0.019). The PetCO2 levels of mortality group at the 5th, 10th, 15th and 20th minutes were found to be meaningfully lower as compared to the ROSC group (p<0.001) (Table-III).

**Table-III:** Followed-Up PetCO2 Levels in ROSC and Exitus Groups

*Follow-Up Time*	*ROSC *	*Exitus *	*p-value * ^a^
PetCO_2_ 0th min	28 (14-99)	24 (3-82)	0.058
PetCO2 5th min	29 (11-84)	20 (4-55)	<0.001
PetCO2 10th min	34 (7-85)	19 (4-50)	<0.001
PetCO2 15th min	34,5 (14-60)	18 (6-50)	<0.001
PetCO2 20th min	34 (12-69)	18 (4-52)	<0.001
PetCO2 25th min	39 (12-60)	18 (8-40)	0.033
PetCO2 30th min	28 (20-66)	17 (10-32)	0.019
PetCO2 35th min	-	16 (8-29)	-
PetCO2 40th min	-	16 (10-24)	-

a: Results for p<0.0071 according to Bonferroni Correction were accepted as statistically significant

**Fig.2:**
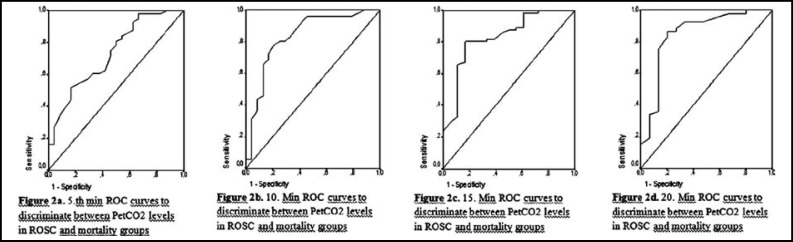
ROC curves to discriminate between PetCO2 levels in ROCS and mortality groups

In distinguishing ROSC and mortality groups, the 5th minute measurements of PetCO2 were statistically significant differentiator and the area below the ROC curve was determined to be 0.730 (95% Confidence Interval: 0.610-0.849) and the best intersection point was 20.5 (p<0.001). In distinguishing ROSC and mortality groups, the 10th minute measurements of PetCO2 were statistically significant differentiator and the area below the ROC curve was determined to be 0.836 (95% Confidence Interval: 0.730-0.941) and the best intersection point was 24.5 (p<0.001). In distinguishing ROSC and mortality groups, the 15th minute measurements of PetCO2 were statistically meaningful differentiator and the area below the ROC curve was determined to be 0.827 (95% Confidence Interval: 0.714-0.940) and the best intersection point was 25.5 (p<0.001). In distinguishing ROSC and mortality groups, the 20th minute measurements of PetCO2 were statistically significant differentiator and the area below the ROC curve was determined to be 0.850 (95% Confidence Interval: 0.721-0.980) and the best intersection point was 28.0 (p<0.001) (Fig.2).

In distinguishing ROSC and mortality, the diagnostic performance indicators of PetCO2 measurements increased cumulatively as they progress from the 5th minute to the 20th. In distinguishing ROSC and mortality, among the measurements between the 5th and 20th minutes, PetCO2 exhibits the highest performance at the 20th and the lowest at the 5th minute. PetCO2 sensitivity, specificity, positive and negative estimate values, and linearity ratios of PetCO2 within the measurements taken in the 5-20 minute interval are given in Table-IV.

**Table-IV:** Diagnostic Performance Indicators on Best Intersection Point of 5th, 10th, 15th and 20th min PetCO2 Measurements in Differentiating ROSC and Exitus Groups

*Indicators*	*Definitions*	*PetCO2 5th min*	*PetCO2 10th min*	*PetCO2 15th min*	*PetCO2* *20th min*
Number of patients	N	80	79	73	68
Sensitivity	TP/(TP+FN)	29/56 (%51.8)	43/55 (%78.2)	44/55 (%80.0)	46/53 (%86.8)
Specificity	TN/(TN+FP)	20/24 (%83.3)	19/24 (%79.2)	15/18 (%83.3)	12/15 (%80.0)
PPV	TP/(TP+FP)	29/33 (%87.9)	43/48 (%89.6)	44/47 (%93.6)	46/49 (%93.9)
NPV	TN/(FN+TN)	20/47 (%42.6)	19/31 (%61.3)	15/26 (%57.7)	12/19 (%63.2)
Accuracy	(TP+TN)/(N)	49/80 (%)	62/79 (%)	59/73 (%)	58/68 (%)
p value		0.003	<0.001	<0.001	<0.001

TP: True Positive, FN: False Negative, TN: True Negative, FP: False Positive,

PEV: Positive Predicted Value, NEV: Negative Predicted Value.

## DISCUSSION

Initial and final PetCO2 values of patients were found respectively as 24.5 (3-99) and 20 (4-75) mmHg. We found the PetCO2 level in the 5th, 10th, 15th and 20th minutes to be significantly higher in the ROSC group as compared to the mortality group (p<0.001). In differentiating ROSC and mortality, PetCO2 exhibited highest performance at the 20th minute and the lowest at 5th among the measurements taken in the 5-20th minute interval. We determined the statistically significant distinguishing area as 0.850 (%95 Confidence Interval: 0.721-0.980) in PetCO2 20th minute measurements and the best intersection point as 28.0 mmHg (p<0.001). There was no statistically significant difference observed between the initial presenting rhythms and PetCO2 levels of the patients (p>0.05). Also, none of the patients who had PetCO2 levels less than 14 mmHg survived.

Findings related to positive correlation between cardiac output and PetCO2 had resulted in widespread use of capnography in cardiac arrest situations.^4^^,^^9^ When chest compressions are ceased during the CPR, the PetCO2 values are seen to decrease to zero after a short period of time. The increase in the pulmonary perfusion followed by the initiation chest compressions result in increased PetCO2 values. Therefore, PetCO2 is thought to be a good indicator for the quality of the CPR. A rapid increase in PetCO2 value is thought to show the ROSC.^10^^,^^11^

A number of animal and human studies have shown an excellent correlation between PetCO2 and cardiac output during cardiopulmonary resuscitation and during states of low blood flow, making capnometry an effective tool to assist in evaluating the efficacy of cardiopulmonary resuscitation efforts.^12^ A previous study looked at this question in 34 patients, 9 of whom survived resuscitation. These 9 patients had higher average PetCO2 levels during CPR than the other 25.^13^ Other studies confirmed these findings through different methodologies. In one study, Levine et al^14^ evaluated 150 consecutive victims of cardiac arrest outside the hospital who had pulseless electrical activity. The patients were intubated and evaluated by mainstream PetCO2 monitoring. The authors’ hypothesis was that a PetCO2 level of 10 mmHg or less after 20 minutes of CPR would be predictive of death. Of the 150 patients, 35 patients survived to hospital admission, and in fact, the study showed that after 20 minutes of CPR, a PetCO2 value of 10 mmHg or less was predictive of death.^14^ Whereas in our study we determined this value to be 18 mmHg on average. Initial PetCO2 value measured in all ROSC patients was 34 mmHg on the average. During the CPR period, PetCO2 levels of mortality group progressively decreases. The most reliable interval is 20th minute similar to the above studies.

In the first of two studies which investigated whether the initial PetCO2 measurement at pre-hospitalization CPR might provide an indicator for survival, only one case with PetCO2 value below 10 mmHg out of 127 survived, and none out of 139 cases in the second study.^15^^,^^16^ According to our results, none of the patients with PetCO2 value below 14 mmHg survived.

In the study by Grmec et al, the pre-hospital PetCO2 levels of 44 asphyxial cardiac arrest patients (in PEA or asystole) and 141 primary cardiac arrest patients (in VF or pulseless VT) were compared.^16^ They found PetCO2 values measured during the 1st minute of CPR in the cardiac arrest group and suggested that arrest etiology may be differentiated on the basis of 1st minute values. Whereas in our study no statistically significant difference was observed in terms of average PetCO2 levels between the VF arrival rhythm group and PEA/Asystole group (p=0.519). This conflicting variation might have been caused by transportation times to the emergency department. During the asphyxia period, the pre-cardiac arrest CO2 is still being produced in the lungs. This causes the level of CO2 to be high in the exhaled breath.^6^ In our study, the average transport time of patients to the emergency department was 15 minutes and none of them had their PetCO2 levels measure during the transport. For this reason, probable PetCO2 value differences which might be resulting from different arrest etiologies (asphyxia and cardiac) could not be determined.

## CONCLUSION

According to our research, PetCO2 values are higher in the ROSC group. During the CPR, the most reliable time for ROSC estimation according to PetCO2 values is 20th minute. None of the patients who had PetCO2 levels less than 14 mmHg survived. There was no statistically significant difference observed between the initial presenting rhythms and PetCO2 levels of the patients.

## Authors contribution

Emine Akıncı conceived, designed, manuscript writing and did editing of manuscript

Hayri Ramadan did data collection

Yücel Yüzbaşıoğlu did data collection

Figen Coşkun did review and final approval of manuscript

## References

[1] Kramer-Johansen J, Myklebust H, Wik L, Fellows B, Svensson L, Sorebo H (2006). Quality of out-of-hospital cardiopulmonary resuscitation with real time automated feedback: a prospective interventional study. Resuscitation.

[2] Edelson DP, Litzinger B, Arora V, Walsh D, Kim S, Lauderdale DS (2008). Improving in-hospital cardiac arrest process and outcomes with performance debriefing. Arch Intern Med.

[3] White RD, Asplin BR (1994). Out-of-hospital quantitative monitoring of end-tidal carbon dioxide pressure during CPR. Ann Emerg Med.

[4] Ornato JP, Garnett AR, Glauser FL (1990). Relationship between cardiac output and the end-tidal carbon dioxide tension. Ann Emerg Med.

[5] Garnett AR, Ornato JP, Gonzalez ER, Johnson EB (1987). End-tidal carbon dioxide monitoring during cardiopulmonary resuscitation. JAMA.

[6] Bhende MS, Karasic DG, Karasic RB (1996). End-tidal carbon dioxide changes during cardiopulmonary resuscitation after experimental asphyxial cardiac arrest. Am J Emerg Med.

[7] Berg RA, Hemphill R, Abella BS, Aufderheide TP, Cave DM, Hazinski MF (2010). Part 5: Adult Basic Life Support: 2010 American Heart Association Guidelines for Cardiopulmonary Resuscitation and Emergency Cardiovascular Care. Circulation.

[8] Neumar RW, Otto CW, Link MS, Kronick SL, Shuster M, Callaway CW (2010). Part 8: adult advanced cardiovascular life support: 2010 American Heart Association Guidelines for Cardiopulmonary Resuscitation and Emergency Cardiovascular Care. Circulation.

[9] White RD, Asplin BR (1994). Out-of-hospital quantitative monitoring of end-tidal carbon dioxide pressure during CPR. Ann Emerg Med.

[10] Entholzner E, Felber A, Mielke L, Hargasser S, Breinbauer B, von Hundelshausen B (1992). The determination of end-expiratory CO2 during resuscitation. Experience and results with the Normocap 200 (Fa. Datex) in preclinical resuscitation conditions. Anasthesiol Intensivmed Notfallmed Schmerzther.

[11] Bhende MS, Karasic DG, Karasic RB (1996). End-tidal carbon dioxide changes during cardiopulmonary resuscitation after experimental asphyxial cardiac arrest. Am J Emerg Med.

[12] Cone DC, Cahill JC, Wayne MA, Gravenstein JS (2004). Cardiopulmonary resuscitation. Capnography: Clinical Aspects.

[13] Sanders AB, Ewy GA (1985). Expired CO2 as a prognostic indicator of successful resuscitation from cardiac arrest. Ann Emerg Med.

[14] Levine RL, Wayne MA, Miller CC (1997). End-tidal carbon dioxide and outcome of out-of-hospital cardiac arrest. NEJM.

[15] Ahrens T, Schallom L, Bettorf K, Ellner S, Hurt G, O’Mara V (2001). End-tidal carbon dioxide measurements as a prognostic indicator of outcome in cardiac arrest. Am J Crit Care.

[16] Grmec S, Klemen P ( 2001). Does the end-tidal carbon dioxide (EtCO2) concentration have prognostic value during out-of-hospital cardiac arrest?. Eur J Emerg Med.

